# *Notes from the Field*: Multiple Cases of Seoul Virus Infection in a Household with Infected Pet Rats — Tennessee, December 2016–April 2017

**DOI:** 10.15585/mmwr.mm6640a4

**Published:** 2017-10-13

**Authors:** Mary-Margaret A. Fill, Heather Mullins, Andrew Stephen May, Heather Henderson, Shelley M. Brown, Cheng-Feng Chiang, Nishi R. Patel, John D. Klena, Annabelle de St. Maurice, Barbara Knust, Stuart T. Nichol, John R. Dunn, William Schaffner, Timothy F. Jones

**Affiliations:** ^1^ Division of Communicable and Environmental Diseases and Emergency Preparedness, Tennessee Department of Health; ^2^Sullivan County Regional Health Department, Blountville, Tennessee; ^3^Viral Special Pathogens Branch, Division of High Consequence Pathogens and Pathology, National Center for Emerging and Zoonotic Diseases, CDC; ^4^Epidemic Intelligence Service, CDC; ^5^Department of Health Policy, Vanderbilt University School of Medicine, Nashville, Tennessee.

In late December 2016, a female aged 18 years in Tennessee (patient A) developed fever, chills, anorexia, nausea, and hematuria. Approximately 1 week later, she was evaluated by her local physician and received a diagnosis of an unspecified viral illness. Laboratory testing at that time was notable only for an elevated creatinine level (1.27 mg/dL; normal = 0.60–1.10 mg/dL). She recovered from her illness without treatment or complications.

In January 2017, an outbreak of Seoul virus infection was identified among rat breeders and owners in Wisconsin and Illinois. CDC assisted Illinois and Wisconsin health officials in performing tracing of potentially infected or exposed rats, and in late January 2017, the Tennessee Department of Health was notified that pet rats owned by patient A were linked to confirmed Seoul virus–infected rats. On February 14, 2017, a follow-up specimen of patient A’s blood tested positive for Seoul virus immunoglobulin M and immunoglobulin G by enzyme-linked immunosorbent assay; she declined testing of her rats, although they were presumed to be positive in light of the patient’s confirmed infection. Consistent with CDC guidance, the Tennessee Department of Health recommended euthanizing the rats; however, patient A refused. In collaboration with the Tennessee Department of Agriculture, an order of quarantine was issued to patient A, prohibiting movement of the rodents from her home. In addition, she and her family received extensive education about risk reduction techniques, including avoiding contact with rodent urine, droppings, saliva, and nesting materials.

In late April 2017, patient B, aged 38 years and the mother of patient A, was evaluated at a local hospital emergency department for multiple days of high fever, anorexia, fatigue, and shortness of breath. At the time of evaluation, her fever was 104.5°F (40.3°C). Laboratory testing was notable for a slightly elevated creatinine level (1.13 mg/dL) and slight thrombocytopenia (platelet count = 143,000/*μ*L; normal = 150,000–450,000/*μ*L). A blood specimen tested positive for Seoul virus immunoglobulin M and immunoglobulin G by enzyme-linked immunosorbent assay, and Seoul virus RNA was detected by reverse transcription–polymerase chain reaction testing. Although patient B lived in the same house as patient A, she only recalled one noteworthy exposure to rodent droppings, having cleaned some from a bathtub approximately 3 weeks before her illness onset.

Seoul virus is a rodent-borne hantavirus, which has been associated with hemorrhagic fever with renal syndrome,* and has a mortality rate of approximately 1–2% (*1*). It is transmitted by the brown Norway rat (*Rattus norvegicus*), which is found worldwide (*2*,*3*). As of January 2017, 17 laboratory-confirmed acute human cases of Seoul virus infection associated with pet rat contact have been identified in the United States as part of this outbreak investigation (*4*). To prevent human cases of Seoul virus infection, CDC recommends testing pet rats for Seoul virus infection and taking measures to avoid unprotected contact with infected rats or their urine, droppings, saliva, and nesting materials, and after safe cleaning techniques (*1*). Euthanasia is recommended for rats with Seoul virus infection; however, this guidance might not be heeded. For certain persons, such as rat breeders, these animals are a financial investment; for others, they are personal pets with which owners might have a substantial emotional attachment. Owners who choose to keep a potentially infected rodent place themselves, other household members, and visitors at risk for infection. Additionally, Seoul virus is easily transmitted within breeding colonies of rats, further propagating the virus and risk for human illness. This report demonstrates ongoing risk for Seoul virus infection for persons living in or visiting households with Seoul virus–infected rodents. Adherence to recommendations for euthanasia will help to mitigate ongoing risk for Seoul virus morbidity and mortality in humans.

## Footnotes

* https://www.cdc.gov/hantavirus/hfrs/index.html.
